# Dicodon monitoring of protein synthesis (DiCoMPS) reveals levels of synthesis of a viral protein in single cells

**DOI:** 10.1093/nar/gkt686

**Published:** 2013-08-20

**Authors:** Sima Barhoom, Ian Farrell, Ben Shai, Dvir Dahary, Barry S. Cooperman, Zeev Smilansky, Orna Elroy-Stein, Marcelo Ehrlich

**Affiliations:** ^1^Department of Cell Research and Immunology, George S. Wise Faculty of Life Sciences, Tel Aviv University, Tel Aviv 69978, Israel, ^2^Anima Cell Metrology, Inc., Bernardsville, NJ 07924-2270, USA and ^3^Department of Chemistry, University of Pennsylvania, Philadelphia, PA 19104-6323, USA

## Abstract

The current report represents a further advancement of our previously reported technology termed Fluorescent transfer RNA (tRNA) for Translation Monitoring (FtTM), for monitoring of active global protein synthesis sites in single live cells. FtTM measures Förster resonance energy transfer (FRET) signals, generated when fluorescent tRNAs (fl-tRNAs), separately labeled as a FRET pair, occupy adjacent sites on the ribosome. The current technology, termed DiCodon Monitoring of Protein Synthesis (DiCoMPS), was developed for monitoring active synthesis of a specific protein. In DiCoMPS, specific fl-tRNA pair combinations are selected for transfection, based on the degree of enrichment of a dicodon sequence to which they bind in the mRNA of interest, relative to the background transcriptome of the cell in which the assay is performed. In this study, we used cells infected with the Epizootic Hemorrhagic Disease Virus 2-Ibaraki and measured, through DiCoMPS, the synthesis of the viral non-structural protein 3 (NS3), which is enriched in the AUA:AUA dicodon. fl-tRNA^Ile^_UAU_-generated FRET signals were specifically enhanced in infected cells, increased in the course of infection and were diminished on siRNA-mediated knockdown of NS3. Our results establish an experimental approach for the single-cell measurement of the levels of synthesis of a specific viral protein.

## INTRODUCTION

It is now accepted that regulation of protein levels has a central posttranscriptional component (1–3). Indeed, mRNA levels are poor predictors of the cellular proteome. Translational control was recently shown to play an important role in cellular responses to stress, apoptosis and viral infections (4–6). These new insights rely on data produced by two novel measurement techniques: at the cell population level, precise measurements of protein quantities using mass spectrometry and, at the individual cell level, quantification of specific proteins using high-throughput imaging methods. However, steady-state protein levels do not directly report on the rates of either protein synthesis or degradation, two processes that may be regulated independently of one another. A general method for measuring the rates of synthesis of specific proteins in single cells is currently unavailable.

Recently, approaches for monitoring ongoing total protein synthesis in single cells that do not require genetic manipulation were developed. Puromycin (or its derivatives) has been used to label and image nascent proteins in cells as a measure of active translation (7–12). Another methodology uses Fluorescent transfer RNA (tRNA) for Translation Monitoring (FtTM), as described in our previous report (13). In FtTM, which is based on the transfection of bulk fluorescently labeled tRNAs, a Förster Resonance Energy Transfer (FRET) signal is generated when a donor-labeled tRNA binds next to an acceptor-labeled tRNA in adjacent sites on an active ribosome and is imaged by fluorescence microscopy. Using FtTM, we measured global alterations to protein synthesis such as its decrease on ER-stress, enhancement on activation of astrocytes and altered compartmentalization in cells infected with the Epizootic Hemorrhagic Disease Virus (EHDV) (13).

In contrast to puromycin, tRNAs are intrinsically sequence specific, creating the potential for detection and measurement of specific isoacceptor tRNA pairs bound to ribosomes that are cognate to specific mRNA dicodons. Exploiting such potential requires transfection of cells with specific isoacceptor fluorescent tRNAs (fl-tRNAs), rather than bulk fl-tRNAs. Here, we apply a newly developed extension of the FtTM approach, termed DiCodon Monitoring of Protein Synthesis (DiCoMPS), to monitor the translation of a specific viral mRNA within fixed infected Chinese hamster ovary (CHO) and Ovine Kidney (OK) cells, a key step for elucidating the complex nature of viral-host interactions. Viral infections provide a favorable setting for demonstrating the feasibility of the DiCoMPS approach. Viruses depend strictly on host protein synthesis machinery for propagation. Regulation of the host mRNA translation machinery by the viral infection process (‘hijacking’) is central to virus-host interactions, involving the activation of cellular anti-viral mechanisms and their usurpation by the virus (5,6). For example, in addition to the re-compartmentalization of protein synthesis, EHDV infection induces activation of the ds-RNA dependent protein kinase, the phosphorylation of eukaryotic initiation factor 2α (eIF2α), and induction of autophagy (Shai *et al.*, submitted manuscript). These processes can affect the repertoire of translated transcripts in the infected cells. Monitoring the rates of synthesis of specific proteins during the course of infection can help to elucidate the complex nature of viral-host interactions. Here, we demonstrate, for the first time, the use of the DiCoMPS approach to specifically measure the synthesis of the non-structural viral protein non-structural protein 3 (NS3) in single EHDV-infected cells. In doing so, we use a specific tRNA isoacceptor pair, cognate to a dicodon that is highly enriched within the mRNA encoding NS3 relative to its occurrence within the host mRNA transcriptome.

## MATERIALS AND METHODS

### Cell cultures and virus

Spontaneously immortalized OK cells were generated at the Kimron Veterinary Institute, Beit-Dagan, Israel and were a kind gift of Dr Hagai Yadin; CHO cells were from ATCC. Cells were maintained in Modified Eagle's Medium (MEM) (OK) or F-12 (CHO) supplemented with 10% (v/v) fetal calf serum, 5 mM l-glutamine and antibiotics (all from Biological Industries Ltd). The EHDV2-Ibaraki Virus was obtained from Dr T. Tsuda, of the Kyushu Research Station, National Institute of Animal Health, Chuzan, Kagoshima, Japan and donated for this study by Dr Hagai Yadin of the Kimron Veterinary Institute, Beit Dagan, Israel.

### Virus purification

EHDV2-IBAV-infected OK cells were collected 48 h post infection and pelleted at 4°C. The pellet was resuspended in 6 ml of TNET Buffer [50 mM Tris–HCl (pH 8.0), 0.2 M NaCl, 5 mM EDTA, 0.5% Triton X-100] and homogenized (10 strokes) using a Glass homogenizer (7 ml). The homogenate was layered onto a sucrose cushion comprising 66 and 40% sucrose each prepared in 0.2 M Tris. Samples were centrifuged in a Beckman Ultracentrifuge using a SW41 Rotor, at 23 000 r.p.m., 4°C for 3 h. Purified virus was extracted from the interface of the sucrose cushions and 10 mM dithiothreitol was added to prevent virus aggregation.

### Virus infection for microscopy assays

CHO or OK cells, seeded on glass coverslips at 1 × 10^5^ and at 7 × 10^4^ cells/well, respectively, in 24-well plates, were infected with EHDV2-IBA [multiplicity of infecton (MOI) = 2.3–4 per cell] for different periods. Infected cells were subsequently transfected with bulk or specific labeled tRNAs (see under Transfection protocol and FRET assay protocol).

### Plaque assay

OK cells were seeded at 1.5 × 10^5^ cells/well in 6-well plates, transfected with siRNA NS3 mix or siRNA scrambled (according to manufacturer’s protocol, see siRNA silencing assay) and infected with EHDV2-IBA (MOI = 2.3–4 per cell) at 7 h posttransfection. The medium was replaced 4 h post infection. After an additional 12 h, virus was extracted from the collected growth medium or from the cells by sonication. Sequential 10-fold dilutions of EHDV2-IBA were prepared in MEM and used to infect OK cells seeded at 60–70% confluence in 12-well plate (500 µl of each dilution). An uninfected well was used as control. Plates were incubated at 37°C for 1 h; cells were washed and overlayed with 0.3% tragacanth (Sigma-Aldrich, St. Louis, MO, USA) in MEM. Fixation (after 2–4 days) was with crystal violet (Sigma-Aldrich, St. Louis, MO, USA)/formaldehyde.

### Antibodies

Monoclonal mouse anti-non-structural protein 2 (NS2) antibodies were generated with the following peptide: n′–PEPKGYVLEISEVGSYRIQDG–c′ (13). For the production of polyclonal rabbit- and monoclonal mouse anti-NS3 antibodies, mice and rabbits were immunized with the following peptide: n′-CDEMSLVPYQENVRPPS-c′ (corresponding to amino acids 19–34 of EHDV2-IBA NS3, GeneScript Corporation (NJ, USA). Mouse anti-α-Tubulin was from BioLegend Inc. (San Diego, CA, USA). Alexa 647-conjugated secondary antibodies were from Molecular Probes (Eugene, OR, USA); horseradish peroxidase-conjugated secondary antibodies were from Jackson ImmunoResearch Laboratories (West Grove, PA, USA). 4′,6-diamidino-2-phenylindole (DAPI) was from Sigma-Aldrich (St. Louis, Mo, USA).

### Immunofluorescence

OK or CHO cells, pre- or post-viral infection and/or transfection, were fixed (4% paraformaldehyde, 30 min, room temperature), permeabilized [0.1% Triton-X in phosphate buffered saline (PBS), 10 min], extensively washed with PBS, blocked [2% bovine serum albumin, bovine serum albumin (BSA) in PBS (PBS/BSA), 1 h] and stained using polyclonal rabbit anti-NS3 antibodies at 1:500 dilution (2 h in PBS/BSA, room temperature) and the secondary antibodies AlexaFluor-647 IgG goat anti-rabbit (Invitrogen; 1:500 dilution, PBS/BSA, 1 h, room temperature). Coverslips were mounted with Fluoromount (Sigma).

### Protein synthesis measurements and immunobloting

OK or CHO cells, infected or not with EHDV2-IBA for different time points, treated or not with puromycin (1 mM, 10 min, Mercury), were labeled [30 min, in 0.3 ml of methionine–cysteine-free Dulbecco’s modiﬁed Eagle’s medium supplemented with: 2 mM l-glutamine, 10% dialyzed fetal calf serum (Sigma), 15 μCi/ml of [^35^S]-L-methionine and [^35^S]-L-cysteine]. Cells were harvested following addition of 0.5 ml of cold PBS containing 100 μg/ml cycloheximide and washed twice with cold PBS. The global protein synthesis rate was determined as described (14). For assessment of the pattern of synthesized proteins in cells, 5 µg of total proteins were separated by 12% sodium-dodecyl-sulfate polyacrilamide gel electrophoresis (SDS–PAGE), transferred to nitrocellulose membrane and [^35^S]-Met/Cys incorporation was visualized with a Phosphor-Imager. The densitometric pattern was determined with ImageJ software. The same membrane was also washed, blocked and incubated with anti-NS3 antibody or anti-tubulin and visualized using enhanced chemiluminescence (ECL, Amersham). For the assessment of NS3 during viral infection, 10 µg of proteins from infected and non-infected cells were separated by 12% SDS–PAGE.

### tRNA

*Isoacceptor tRNA purification and characterization.* tRNA^Ile^_UAU_, tRNA^Gly^_CCC_ and tRNA^Pro^_AGG_ were isolated from bulk yeast tRNA (Roche Diagnostics) in an optimized procedure similar to that described (15). Streptavidin-linked agarose beads (SA beads, Sigma Aldrich) were incubated with 3′-biotin labeled oligoDNAs complementary to the D-loop and anticodon loop of tRNA^Ile^_UAU_ (5′-ATAAGCACGAAGCTCTAACCACTGAG-3′-Biotin), tRNA^Gly^_CCC_ (5′-GGGAAGCATGAATTCTAACCACAGAAC-3′-Biotin) and tRNA^Pro^_AGG_ (5′-CCTAAGCGAGAATCATACCTCTAGAC-3′-Biotin) at a ratio of 3:1 (24 nmol binding capacity to 8 nmol DNA oligo) at room temperature for 30 min with shaking in the top cup of an Ultrafree-MC filter tube [10 mM Tris–HCl (pH 7.6)]. The SA beads were collected by centrifugation at 8000 RCF for 1 min and washed two times with 400 μl of 10 mM Tris–HCl (pH 7.6), a procedure that removes unbound oligoDNA (always <15% of the total). Bulk yeast tRNA (200 nmol, 110 A_260_ units) in a total volume of 300 μl of Diethylpyrocarbonate (DEPC) water was heated to 80°C for 10 min followed by rapid addition of 300 µl of room temperature tetramethylammonium chloride buffer [20 mM Tris (pH 7.6), 1.8 M tetramethylammonium, 0.2 mM Ethylenediaminetetraacetic acid (EDTA)]. The resulting solution was added to the oligo bound SA beads and incubated at 65°C for 10 min, followed by a slow decrease in temperature to 37°C (∼15–20 min) and continued incubation at 37°C for an additional 30 min. Unbound tRNAs were removed by centrifugation, and the beads were repeatedly washed with 400 µl of portions of 10 mM Tris–HCl (pH 7.6) at room temperature until the absorbance at 260 nm of the washed solution was <0.1 A_260_ unit/ml (∼7 washes). The washed beads were suspended in 400 μl of 10 mM Tris–HCl (pH 7.6) and target isoacceptor tRNA was eluted by incubating at 55°C (tRNA^Ile^_UAU_ and tRNA^Pro^_AGG_), or 45°C (tRNA^Gly^_CCC_), for 7 min, followed by centrifugation at room temperature in tubes containing 2 μl of 1 M MgCl_2_, which aids proper tRNA folding. The tRNA in the supernatant was then deacylated by incubation with 100 mM Tris–HCl (pH 9.0) at 37°C for 3 h.

Analytical scale aminoacylation with specific radioactive amino acids was performed on purified, deacylated isoacceptor tRNA using bulk yeast aminoacyl synthetase for tRNA^Ile^ and tRNA^Gly^ as described (16). For tRNA^Pro^, 3′-CCA end repair was necessary and performed by incubating tRNA^Pro^ (10 μM, 200 pmol) at 80°C for 3 min, followed by addition of 4 μl of repair buffer containing 500 mM glycine, 50 mM MgCl_2_ and 5 mM dithiothreitol and incubation at room temperature for 10 min. ATP (1 mM), CTP (1 mM) and CCA repair enzyme from *Escherichia coli* (5 μM) was added to the mixture to a final volume of 20 μl and incubated at 37°C for 30 min. After incubation, the final volume was increased to 400 μl with water, and a phenol extraction was performed followed by an ethanol precipitation. The tRNA was redissolved in 400 μl of water and passed through a YM-10 (10 000 Da cutoff) filter tube two times to remove any excess ATP or CTP. A purified proline aminoacyl synthetase for tRNA^Pro^ (final concentration 0.4 μM), prepared as described (17), was used for aminoacylation after end repair. The analytical scale aminoacylation resulted in the following aminoacylation efficiencies: 0.68 Ile/tRNA^Ile^_UAU_, 0.56 Gly/tRNA^Gly^_CCC_ and 0.70 Pro/tRNA^Pro^_AGG_.

RNase T1 digestion and matrix-assisted laser desorption/ionization (MALDI) mass spectrometric analysis was performed on tRNA^Ile^_UAU_ as described (18). MALDI peaks were observed corresponding to oligonucleotides present in tRNA^Ile^_UAU_ but absent in the other Ile isoacceptor, tRNA^Ile^_AAU_ (UALp, CUCGp, CACCAp, CUPCGp, ACC7DGp, CUPAP6ACGp). In addition, no MALDI peaks were observed for oligonucleotides present in tRNA^Ile^_AAU_ but absent in tRNA^Ile^_UAU_ (CUIp, DDGp, TPCGp, CUAGp, DDAAGp, ADAGp, UCCCGp, AU6ACG, ACCACCAp, UCUCUUKLp), whereas these latter peaks were observed on analysis of RNase T1 digests of tRNA^Ile^_AAU_. The one symbol code for modified bases is as follows: l: N2-methylguanosine, d: dihydrouridine, P: pseudouridine, 7: 7-methylguanosine, 5: 5-methylcytidine, 6: N6-methyl-N6-threonylcarbomoyladenosine, I: inosine, T: thymine, K: 1-methylguanosine.

*Fluorescent labeling.* Dihydrouridines present in bulk or purified isoacceptor tRNAs were reduced with NaBH_4_ and labeled with either Rhodamine 110 (Rd110) or Cy3-hydrazide as described (16,18). The labeling efficiencies were 0.87 Rd110/bulk tRNA, 0.86 Rd110/tRNA^Ile^_UAU_, 0.68 Rd110/tRNA^Gly^_CCC_, 0.68 Cy3/bulk tRNA, 0.83 Cy3/tRNA^Ile^_UAU_ and 0.48 Cy3/tRNA^Pro^_AGG_.

### siRNA silencing assay

Transfection of siRNA was performed with INTERFERin™ (Polypus) according to manufacture’s instructions. The following siRNA oligos (Dharmacon) were used: α-NS3 combination of - CGGAAATAATACA; and GAAAGGAGATAATGAAGAA and a commercially supplied scrambled sequence (negative control). Transfections were carried out with cells grown on 13 mm coverslips (in 24-well plates) for microscopy-based experiments, in 6-well plates for immunoblotting experiments or in 12-well plates for plaque assays.

### FRET assay

OK or CHO cells were co-transfected with: bulk yeast tRNAs labeled with Rd110- (donor) and/or Cy3- (acceptor), bulk Rd110 and Cy3-tRNA^Ile^_UAU_, Cy3-tRNA^Ile^_UAU_ and Rd110-tRNA^Ile^_UAU_ or with Cy3-tRNA^Pro^_AGG_ and Rd110-tRNA^Gly^_CCC_ tRNAs, fixed 6 h post transfection, immunostained with anti-NS3 antibodies, mounted and imaged with a spinning disc confocal microscope. A raw FRET signal consisting of the recorded emission above 570 nm in response to illumination at 473 nm served as a basis for the calculated FRET signal (FRETc; the FRET signal after the elimination of background and of the non-specific contribution originating from the experimentally measured donor bleed through and direct excitation of the acceptor under the FRET illumination).

### Microscopy

Images were acquired with a spinning disk confocal (Yokogawa CSU-22 Confocal Head) microscope (Axiovert 200 M, Carl Zeiss MicroImaging) under control of SlideBook™ (Intelligent Imaging Innovations). Images were acquired with an Evolve EMCCD camera (Photometrics; 100× lens, 1 × 1 binning, yielding a pixel size of 0.16 microns).

### Bioinformatics

For the selection of the tRNA pairs to be used in DiCoMPs, we devised a strategy based on the calculated estimate of the signal-to-background ratio for the protein of interest (POI, signal) relative to the potential repertoire of proteins encoded in the cells being assayed (background). In this study, we infected OK cells (ovine) and CHO cells (hamster) with EHDV2-IBA. Therefore, such calculation was performed with the 10 proteins encoded by EHDV2-IBA (serving as POIs) against the background of the bovine and mouse transcriptomes. The choice of these transcriptomes stems from the full annotation of the bovine and mouse genomes, from the similarity of the bovine and ovine transcriptomes [∼3% difference at the nucleotide level, (19)] and from the assumed similarity of mouse and hamster transcriptomes. For the calculation of such estimate, we initially assigned a tRNA isoacceptor, expressed in either bovine (for assays with OK cells) or mouse (for assays with CHO cells), to the different codons of the mRNA sequences. Next, based on the dicodons present in the translated regions of these mRNA sequences (POI and transcriptome), we calculated the estimated frequency of tRNA pairs, under the simplifying assumptions of uniform transcript levels for all mRNAs and uniform translation levels. Such frequencies were then used for the calculation of the enrichment factor (E-factor) according to the following formula: E-factor = (frequency of a given tRNA pair in the POI)/(estimated frequency of this same tRNA pair in transcriptome of the species from which the cell originates). The tRNA pairs with E-factors above 50 resulting from such analysis against the bovine transcriptome are presented in Table 1.

**Table 1.** Calculated E-factors of the 10 EHDV2-IBA transcripts relative to the bovine transcriptome

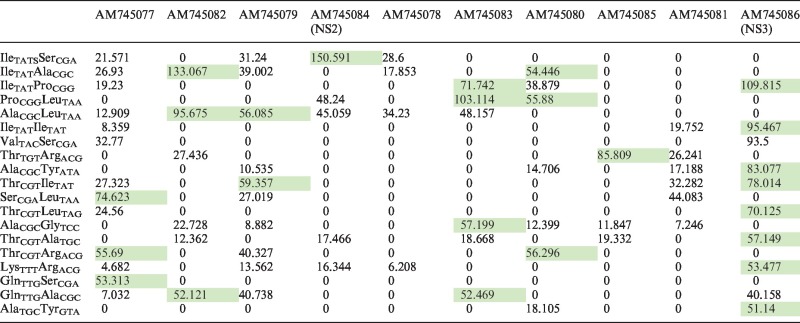

The DiCoMPs approach is based on the calculated estimate of the signal-to-background ratio for the POI relative to the potential repertoire of proteins encoded in the cells being assayed. In this table, such calculation was performed with the 10 proteins encoded by EHDV2-IBA (top row) against the background of the bovine transcriptome. A cognate tRNA isoacceptor, expressed in bovine, was assigned to each codon of the viral or bovine mRNA sequences. Based on the dicodons present in these mRNA sequences, the estimated frequency of tRNA pairs was calculated under the simplifying assumptions of uniform transcript levels for all mRNAs and uniform translation levels. Presented are the enrichment factors (E-factor) for the different EHDV2-IBA proteins with the indicated tRNA pairs (first column), calculated according to the following formula: E-factor = (frequency of the given tRNA pair in the POI)/(estimated average frequency of this same tRNA pair in bovine transcriptome). In this table, the tRNA pairs with E-factors above 50 are highlighted in green.

## RESULTS

**Figure 1.**
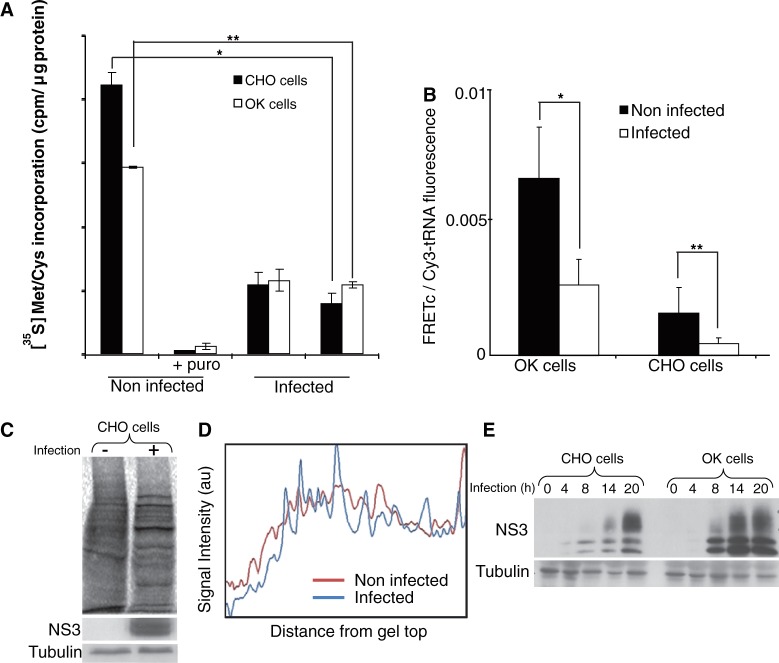
EHDV2-IBA viral infection reduces global protein synthesis and alters the repertoire of synthesized proteins. (**A**) CHO or OK cells infected or not with EHDV2-IBAV (12 or 24 h,) and treated or not with puromycin (1 mM, 10 min), were incubated with [^35^S]-Met/Cys (15 µCi/ml, 30 min), lysed and [^35^S]-Met/Cys incorporation in 20 μg of protein lysate was measured. The graph depicts average ± standard deviation (SD) of three independent experiments done in triplicates. *P < 6E-10, **P < 8E-13. (**B**) CHO or OK cells infected with EHDV2-IBAV (14 h) were co-transfected with yeast bulk Cy3- and Rd110-labeled tRNAs, fixed 6 h post-transfection and imaged by confocal microscopy. FRETc signals were measured and calculated. The graph depicts average of FRETc/Cy3-tRNA fluorescence ratio per cell (n = 25). *P < 9E-9, **P < 0.0001. (**C**) CHO cells, infected or not with EHDV2-IBAV (20 h) and labeled with [^35^S]-Met/Cys (15 µCi/ml, 30 min) were lysed and processed by 10% SDS–PAGE. Nitrocellulose blots were either visualized with phospho-imager or immunoblotted with anti-NS3 or with anti-tubulin antibodies. (**D**) Denistometric analysis of gels depicted in C, carried out with ImageJ. (**E**) CHO or OK cells were infected with EHDV2-IBAV for different time points (4, 8, 14, 20 h). Cells were then lysed and equal amounts of protein were processed by 12% SDS–PAGE and immunoblotted with anti-NS3 or with anti-tubulin antibodies.

The main objective of the present study was to demonstrate the utility of DiCoMPS in monitoring the synthesis of a specific viral protein in an infected cell. For this purpose, we selected CHO cells (broadly used in the biotechnology industry) and OK cells (originating from a natural orbivirus host) for infection with the Ibaraki strain of the Epizootic Hemorrhagic Disease Virus 2 (EHDV2-IBA). To assess whether EHDV2-IBA infection alters the regulation of host protein synthesis, we quantitatively determined overall protein synthesis rates of infected and non-infected cells using measurements that were either cell-population-based ([^35^S]-Met/Cys metabolic labeling) or single-cell-based (bulk-tRNA FtTM) (13). We infected cells at MOIs between 2.3 and 4 (calculated in each experiment by the immunofluorescence-based measurement of the percentage of infected cells at early timepoints of infection). Over this range, 90–98% of cells were infected in all experiments. [^35^S]-Met/Cys incorporation rates showed 2.5–5-fold reductions at both 12 and 24 h postinfection in CHO and OK cells (Figure 1A). Similar reductions were also seen in FRET signals in infected cells as measured by FtTM using bulk-tRNA (Figure 1B). Densitometry analysis of SDS–PAGE separations of ^35^S-labeled lysates of infected and non-infected CHO cells revealed considerable infection-induced changes to the repertoire of synthesized proteins (Figure 1C and D). Analogous results were obtained with OK cells (not shown). Such changes are exemplified by the time-dependent accumulation of the virally encoded NS3, indicative of a productive infection process (Figure 1E). Taken together, these experiments support the notion that EHDV2-IBA induces a partial shutdown of host protein synthesis while manipulating the cell to synthesize proteins required for viral proliferation.

**Figure 2.**
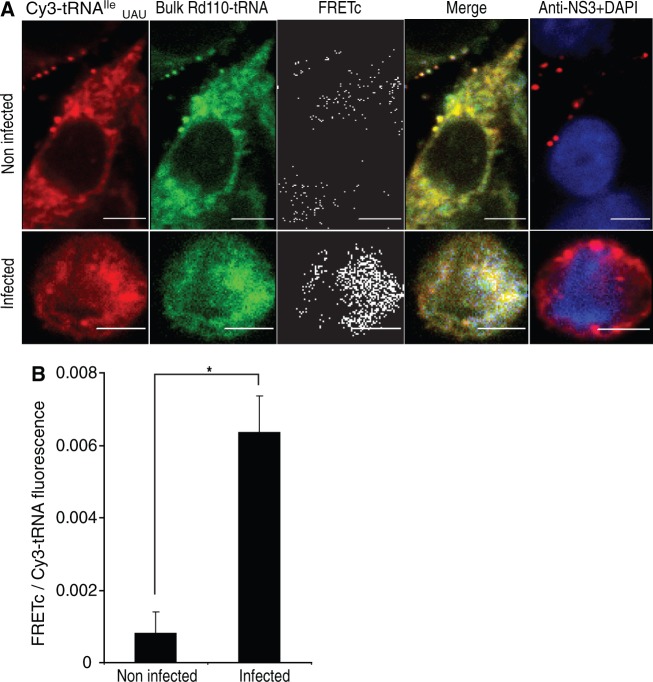
FRET assay with yeast bulk Rd110-tRNA and Cy3-tRNA^Ile^_UAU_ in EHDV2-IBA-infected OK cells. (**A**) OK cells infected with EHDV2-IBAV (14 h) were co-transfected with yeast bulk Rd110-tRNA and Cy3-tRNA^Ile^_UAU_, fixed 6 h posttransfection, immunostained for NS3 and imaged by confocal microscopy (DAPI, blue; NS3, red). Specific FRETc signals were calculated. Panels show representative cells. Bars are 5 µm. (**B**) Graph depicts average ± SD of FRETc/Cy3-tRNA^Ile^_UAU_ fluorescence ratio per cell (n = 27). * *P* < 0.0007.

A comparative analysis of the EHDV2-IBA genome with the coding sequences of 32 randomly selected Ovis Aries cDNA sequences [used in the comparative analysis of bovine and ovine genomes in (19)] uncovered a significant difference in the relative usage of the isoleucine-coding AUA codon (4.2-fold higher in the virus compared with ATA usage in the 32 ovine sequences, *P* < 1 × 10^−^^5^). To test the hypothesis that such a difference in codon usage could be used to characterize changes in the repertoire of translated proteins induced by EHDV2-IBA infection as measured by FRET, OK cells infected or not with EHDV2-IBA, were co-transfected with Rd110-labeled bulk tRNA and Cy3-tRNA^Ile^_UAU_. Using this combination, FRET signals were 8.5-fold higher (*P* = 0.007, Figure 2) in infected cells as compared with non-infected cells, in sharp contrast to the reduction of FRET signals obtained using bulk Rd110-tRNA and bulk Cy3-tRNA with infected cells (Figure 1B).

To further explore the differences in codon usage between the EHDV2-IBA virus and the host cells which it infects, we next used the DiCoMPS approach to monitor the synthesis of viral proteins and of NS3 in particular, during viral infection of both OK and CHO cells. This approach requires identification of a pair of tRNA isoacceptors that are cognate to a dicodon that occurs at much higher frequency within NS3 mRNA and the viral mRNA transcriptome relative to dicodon frequencies within all of the mRNAs encoding the entire host proteome, i.e. the host mRNA transcriptome. The bovine mRNA transcriptome was used for this purpose, as it is fully annotated and predicted to have only an ∼3% difference from the ovine mRNA transcriptome (19). The bovine tRNA table includes 48 isoacceptors (http://gtrnadb.ucsc.edu/) yielding (48^2^–48)/2 + 48 = 1176 distinct possible tRNA pairs. Based on this combination repertoire, we calculated the frequencies of specific tRNA pairs resulting from the translation of viral mRNAs, relative to the corresponding frequencies of these same pairs in the bovine mRNA transcriptome. The ratios between such frequencies, or enrichment factors, are denoted E-factors (see ‘Materials and Methods’ section). The tRNA pairs with E-factors above 50 are shown in Table 1. Of note, the tRNA pair tRNA^Ile^_UAU_: tRNA^Ile^_UAU_ is complementary to a dicodon that appears four times in the EHDV2-IBA mRNA transcriptome (twice in NS3 and once each in the viral proteins VP1 and NS1). The E-factor for this tRNA pair in NS3 is 95.47, arising from the relative rarity of the cognate dicodon in the bovine mRNA transcriptome (72nd rarest of 1176) and its relative abundance in this viral mRNA transcriptome. On the basis of these calculations, we used the labeled tRNA pair tRNA^Ile^_UAU_(Rd110):tRNA^Ile^_UAU_(Cy3) for the measurement of the synthesis rate of EHDV2-IBA proteins in general, and of the NS3 protein in particular. As a negative control, we used tRNA^Gly^_CCC_(Rd110):tRNA^Pro^_AGG_(Cy3), which is complementary to a dicodon that is absent from the viral transcriptome, but appears at a frequency of 0.00186 in the bovine transcriptome (∼4× average dicodon frequency).

**Figure 3.**
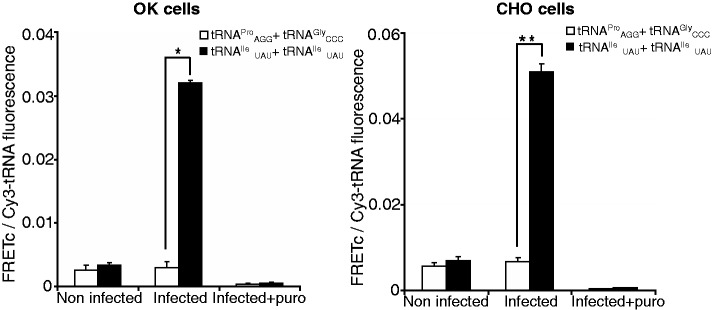
FRET assays with tRNA pairs that match dicodons present (Ile_UAU_ + Ile_UAU_) or absent (Pro_AGG_ + Gly_CCC_) in the EHDV2-IBA genome. CHO or OK cells, infected with EHDV2-IBAV (14 h), were co-transfected either with Cy3-tRNA^Ile^_UAU_ and Rd110-tRNA^Ile^_UAU_ or Cy3-tRNA^Pro^_AGG_ and Rd110-tRNA^Gly^_CCC_, treated or not with puromycin (1 mM, 15 min), fixed 6 h posttransfection, immunostained for NS3 and imaged by confocal microscopy. FRET signals were measured and FRETc signals were calculated. Graphs depict average ± SD of FRETc/Cy3-tRNA fluorescence ratio per cell (n = 30). *P < 3E-18, **P < 2.8E-17.

We initially measured FRET signals generated by both of these fluorescently labeled tRNA pairs in infected and non-infected cells (Figure 3). In both OK and CHO cells, infection with EHDV2-IBA resulted in a marked increase in the FRET signal observed with tRNA^Ile^_UAU_(Rd110):tRNA^Ile^_UAU_(Cy3), in accord with the synthesis of EHDV2-IBA proteins NS3, VP1 and NS1. Importantly, the FRET signal was extinguished by incubation with puromycin (Figure 3), supporting the dependence of this FRET signal on the elongation phase of mRNA translation. In sharp contrast, no such increase in FRET signal on EHDV2-IBA infection was observed in cells transfected with the tRNA^Gly^_CCC_(Rd110):tRNA^Pro^_AGG_(Cy3), which yielded a similarly low signal in both infected and non-infected cells, that was also extinguished by puromycin. Next, we bleached the Cy3-labeled tRNA and measured the changes in Rd110 fluorescence in the bleached cells. In EHDV2-IBA infected cells, we observed increases in Rd110 fluorescence of 12 ± 5% (n = 10, *P* < 2E-5) and 5 ± 3% for cells (n = 7, *P* = 0.002) transfected with tRNA^Ile^_UAU_(Rd110):tRNA^Ile^_UAU_(Cy3) and tRNA^Gly^_CCC_(Rd110):tRNA^Pro^_AGG_(Cy3), respectively, demonstrating that signals arise from energy transfer between donor and acceptor fluorophores.

**Figure 4.**
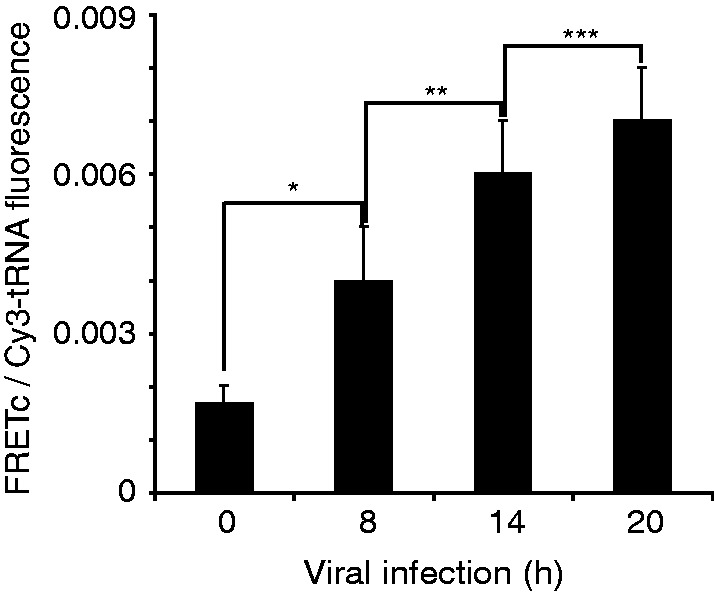
FRET assay with Rd110- and Cy3-labeled tRNA^Ile^_UAU_ reveals signal increase in the course of EHDV2-IBAV infection. OK cells infected with EHDV2-IBAV for different time points (8, 14, 20 h), were co-transfected with Cy3- and Rd110-labeled tRNA^Ile^_UAU_. Cells were fixed 6 h post-transfection, immunostained for NS3 and imaged by confocal microscopy. FRET signals were measured and calculated. The graph depicts average ± SD of FRETc/Cy3-tRNA fluorescence ratio per cell (n = 30 per infection time point). *P < 0.003, **P < 1.1E-5, ***P < 0.002.

EHDV2-IBA infection of both OK and CHO cells is characterized by a progressive rise in NS3 protein content in the infected culture (Figure 1E). Such enhancement can result from either an increase in synthesis or a decrease in degradation. To address this question using DiCoMPS, we infected cells at various time points and transfected them with tRNA^Ile^_UAU_(Rd110):tRNA^Ile^_UAU_(Cy3) 6 h before fixation. The clear increase in FRET signal with infection progression (Figure 4) is in accord with the observed increase in NS3 protein (Figure 1E).

**Figure 5.**
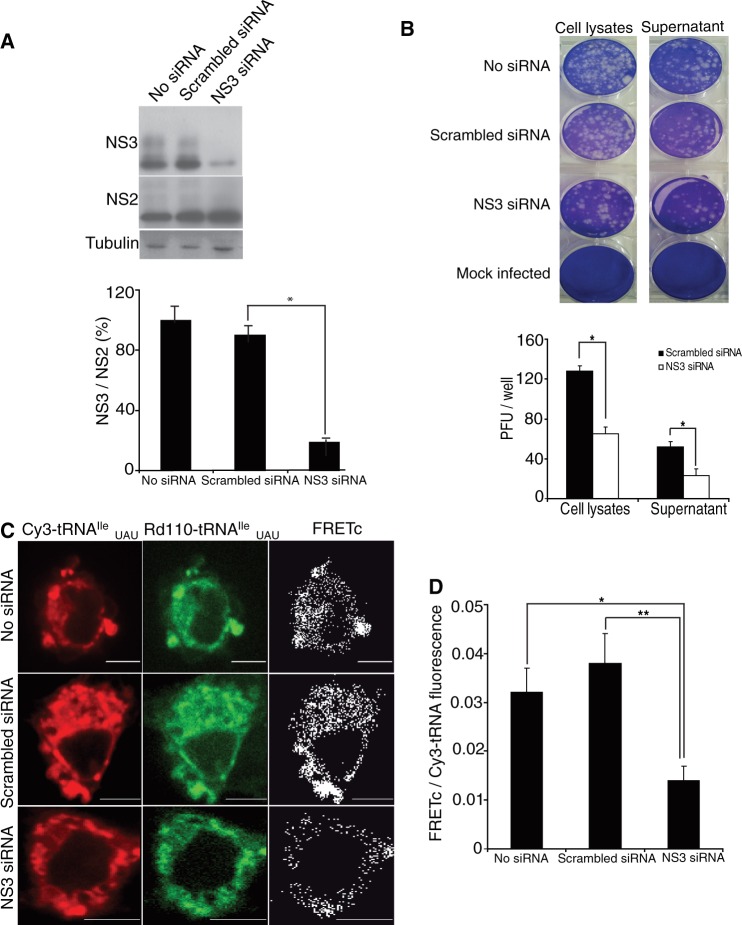
siRNA silencing of NS3. (**A**) OK cells were transfected either with a mix of NS3 specific siRNAs or scrambled control siRNA. Seven hours post-transfection, cells were infected with EHDV2-IBAV for an additional 14 h. Cell lysates were separated by 10% SDS–PAGE and immunoblotted with anti-NS3 or anti-NS2 antibodies. The graph depicts average of ± SD of NS3/NS2 immunoblot signal ratio from four independent experiments. *P < 0.0008. (**B**) OK cells transfected with a mix of NS3 specific siRNAs or scrambled control siRNA were infected with EHDV2-IBAV. 16 h post-infection cell medium was collected, and cells were lysed by sonication. Plaque assay was performed using cell lysates or supernatant. The graph depicts average of plaque forming units (PFU) per well (of 12-well plate) of five independent experiments. *P < 1.2E-11, **P < 1E-10. (**C**) OK cells transfected and infected as the above, were co-transfected with Cy3- and Rd110-labeled tRNA^Ile^_UAU_, fixed 6 h post-tRNA transfection and imaged by confocal microscopy. FRET signals were measured and FRETc signals were calculated. Panels show representative cells. Bars are 5 µm. (**D**) Graph depicts average ± SD of FRETc/Cy3- tRNA fluorescence ratio per cell (n = 30). *P < 1.1E-16, **P < 3E-18.

As mentioned previously, the EHDV2-IBA genome contains four instances of pairs of isoleucine AUA codons (two in NS3 and one each in VP1 and NS1). We used an RNA interference (RNAi) approach to directly measure the contribution of NS3 synthesis to the FRET signal obtained with tRNA^Ile^_UAU_(Rd110):tRNA^Ile^_UAU_(Cy3) in infected cells, using a mix of two oligonucleotides predicted to efficiently reduce NS3 expression (see ‘Materials and Methods’ section). As a prelude to the FRET measurements, we examined the effects of transfection of OK cells with NS3 siRNA (7 h before infection with EHDV2-IBA) both on the overall infection process and on specific virally encoded protein levels. NS3 siRNA transfection resulted in marked and significant reductions in the level of NS3 but had negligible effect on the level of NS2 (Figure 5A), a different non-structural protein, which is also only present in productively infected cells. As determined by plaque assay, NS3 siRNA transfection also resulted in significant reductions in the yield of infective EHDV2-IBA virions, either within the infected cells or present in the cell culture medium (Figure 5B). Importantly, scrambled control siRNA had little effect on either infective virion yield or NS3 level, as compared with untreated infected cells. These results indicate that the initial phases of the infection process proceed under NS3 siRNA transfection, as judged by the synthesis of NS2, even while NS3 synthesis is markedly reduced. On the other hand, the parallel siRNA-mediated reductions in both NS3 synthesis and in the yield of infective EHDV2-IBA suggest that NS3 synthesis rates may directly influence the infection process of EHDV2-IBA.

Next, we examined the effect of NS3 siRNA mix on the FRET signal obtained with tRNA^Ile^_UAU_(Rd110):tRNA^Ile^_UAU_(Cy3) in infected cells. Paralleling the results seen on NS3 synthesis (Figure 5A), a significant reduction in FRET signal intensity (2.9-fold, *P* = 3E-18) was observed on the transfection of the NS3-specific siRNA (Figure 5C and D), whereas no such reduction was observed with the scrambled siRNA. From these results, we can estimate that NS3 synthesis is responsible for ∼60% of the tRNA^Ile^_UAU_(Rd110):tRNA^Ile^_UAU_(Cy3) FRET signal, consistent with the fraction (2 of 4) of AUA:AUA dicodons present in the EHDV-IBA mRNA transcriptome that are present in NS3 mRNA. Overall, the results presented in Figure 5 provide a clear demonstration of the ability of the DiCoMPS approach to measure the synthesis levels of viral protein NS3 in single infected cells.

## DISCUSSION

DiCoMPS represents a significant addition to the existing roster of methods for monitoring protein synthesis in single cells (7,8,10–13,20–22) permitting direct monitoring of translation levels of a target mRNA in genetically unmodified single cells by exploiting the binding of specific tRNA pairs to a specific dicodon within an actively translating ribosome.

Cells infected with EHDV2-IBA are particularly well-suited for application of the DiCoMPS method for three principal reasons. First, the EHDV2-IBA mRNA transcriptome differs in codon usage from that of its host cells (see Table 1), being significantly enriched in the usage of the AUA codon, in general, and of the dicodon AUA-AUA, in particular. Differential codon usage is not limited to EHDV2-IBA. For example, Vaccinia and Influenza A select a repertoire of polysome-associated isoacceptor tRNAs, in accord with selective codon usage, without inducing global alterations to the cellular tRNA repertoire (23). Interestingly, the enhanced use of tRNA^Ile^_UAU_ by EHDV2-IBA may confer an advantage to its replication process, as tRNA^Ile^_UAU_ was unique among other tRNAs in its sensitivity to stimulation of cells with IFN-β and IFN-γ, an integral part of the cellular response to infection (23). Second, EHDV2-IBA infection induces a partial shutdown in host protein synthesis (Figure 1). Such partial shutdown may be elicited by activation of the double-stranded RNA (dsRNA)-activated protein kinase (Shai *et al.*, submitted manuscript) and consists of a characteristic cellular response to many different viral insults (6). Notably, the EHDV2-IBA genome is composed of 10 dsRNA segments that would be expected to induce such anti-viral responses. Third, the infection process is vectorial, allowing for the measurement of an infection-time-dependent increase in viral protein synthesis (Figures 1 and 4).

In addition to demonstrating the feasibility of using DiCoMPS to measure synthesis of specific proteins in single cells, our results also provide important new information concerning the function of NS3 in EHDV2-IBA infection. Although initial assessments of the function of the NS3 protein of the closely related Bluetongue virus suggested a role in viral release (24,25), possibly by functioning as a viroporin (26), a recent study suggests a more complex role in viral maturation and assembly (27). The similarity in the reduction in infective EHDV2-IBA virions that we observed in association with NS3-knockdown cells and in their medium (supernatant) supports the latter suggestion. Alternatively, NS3 siRNA-mediated effects may also stem from alterations in the levels of the S10 genome segment of EHDV2-IBA, a possibility that will be evaluated in future studies. Taken together, our results stress the importance of measuring synthesis levels of specific viral proteins in single cells, as differences in these levels are potential sources of intra-population variability in virion production and in infection-induced cell fate.

Although virally infected cells represent an exceptionally well-suited biological system for DiCoMPS measurements, combining a reduction in the background signal and an augmentation of the protein-specific signal, we expect the DiCoMPS approach will have a more general applicability for measuring the synthesis of specific proteins in cells. We have analyzed the 20 239 human mRNA transcripts in the SwissProt database, and for each one determined the leading tRNA pair and its E-factor with relation to the entire human mRNA transcriptome. Such analysis revealed that 6.3% of proteins have a leading tRNA pair with an E-factor >100, 84.5% have leading tRNA pairs with E-factors ≥ 20 and only 1.2% of proteins have a leading tRNA pair E-factor of < 10. Thus, in cases where the targeted mRNA is undergoing translation in a specific cellular location by a significant fraction of active ribosomes (1–5%), DiCoMPS signals attributable to the synthesis of a specific protein should be clearly discernable. Moreover, the sequence specificity of the DiCoMPS approach may enable the generation of tagged mRNAs of interest for the purpose of analyzing their translation level and localization in single cells. This can be achieved by genetic modification of the target transcripts by fusing specific dicodon tags to the 5′- or 3′- ends of their coding sequence, similarly to the introduction of an epitope tag.

In summary, our results demonstrate the ability of DiCoMPS to report on the synthesis of a specific non-structural viral protein (NS3) in single cells along the course of infection, and partially clarify the function of NS3 in EHDV2-IBA virion production.

## FUNDING

Anima Cell Metrology and by Binational Agricultural Research and Development Fund [BARD, grant No. 15-4192-09; awarded to M.E.]. Funding for open access charge: Anima Cell Metrology.

*Conflict of interest statement.* None declared.

## References

[1] Vogel C, Marcotte EM (2012). Insights into the regulation of protein abundance from proteomic and transcriptomic analyses. Nat. Rev. Genet..

[2] Taniguchi Y, Choi PJ, Li GW, Chen H, Babu M, Hearn J, Emili A, Xie XS (2010). Quantifying *E. coli* proteome and transcriptome with single-molecule sensitivity in single cells. Science.

[3] Maier T, Guell M, Serrano L (2009). Correlation of mRNA and protein in complex biological samples. FEBS Lett..

[4] Holcik M, Sonenberg N (2005). Translational control in stress and apoptosis. Nat. Rev. Mol. Cell. Biol..

[5] Dabo S, Meurs EF (2012). dsRNA-Dependent Protein Kinase PKR and its Role in Stress, Signaling and HCV Infection. Viruses.

[6] Garcia MA, Gil J, Ventoso I, Guerra S, Domingo E, Rivas C, Esteban M (2006). Impact of protein kinase PKR in cell biology: from antiviral to antiproliferative action. Microbiol. Mol. Biol. Rev..

[7] Starck SR, Green HM, Alberola-Ila J, Roberts RW (2004). A general approach to detect protein expression *in vivo* using fluorescent puromycin conjugates. Chem. Biol..

[8] Schmidt EK, Clavarino G, Ceppi M, Pierre P (2009). SUnSET, a nonradioactive method to monitor protein synthesis. Nat. Methods.

[9] Goodman CA, Mabrey DM, Frey JW, Miu MH, Schmidt EK, Pierre P, Hornberger TA (2011). Novel insights into the regulation of skeletal muscle protein synthesis as revealed by a new nonradioactive *in vivo* technique. FASEB J..

[10] David A, Bennink JR, Yewdell JW (2013). Emetine optimally facilitates nascent chain puromycylation and potentiates the ribopuromycylation method (RPM) applied to inert cells. Histochem. Cell Biol..

[11] David A, Dolan BP, Hickman HD, Knowlton JJ, Clavarino G, Pierre P, Bennink JR, Yewdell JW (2012). Nuclear translation visualized by ribosome-bound nascent chain puromycylation. J. Cell. Biol..

[12] Liu J, Xu Y, Stoleru D, Salic A (2012). Imaging protein synthesis in cells and tissues with an alkyne analog of puromycin. Proc. Natl Acad. Sci. USA.

[13] Barhoom S, Kaur J, Cooperman BS, Smorodinsky NI, Smilansky Z, Ehrlich M, Elroy-Stein O (2011). Quantitative single cell monitoring of protein synthesis at subcellular resolution using fluorescently labeled tRNA. Nucleic Acids Res..

[14] Gerlitz G, Jagus R, Elroy-Stein O (2002). Phosphorylation of initiation factor-2 alpha is required for activation of internal translation initiation during cell differentiation. Eur. J. Biochem..

[15] Yokogawa T, Kitamura Y, Nakamura D, Ohno S, Nishikawa K (2010). Optimization of the hybridization-based method for purification of thermostable tRNAs in the presence of tetraalkylammonium salts. Nucleic Acids Res..

[16] Pan D, Qin H, Cooperman BS (2009). Synthesis and functional activity of tRNAs labeled with fluorescent hydrazides in the D-loop. RNA.

[17] SternJohn J, Hati S, Siliciano PG, Musier-Forsyth K (2007). Restoring species-specific posttransfer editing activity to a synthetase with a defunct editing domain. Proc. Natl Acad. Sci. USA.

[18] Kaur J, Raj M, Cooperman BS (2011). Fluorescent labeling of tRNA dihydrouridine residues: Mechanism and distribution. RNA.

[19] Kijas JW, Menzies M, Ingham A (2006). Sequence diversity and rates of molecular evolution between sheep and cattle genes. Anim. Genet..

[20] Rodriguez AJ, Shenoy SM, Singer RH, Condeelis J (2006). Visualization of mRNA translation in living cells. J. Cell. Biol..

[21] Miyata S, Mori Y, Fujiwara T, Ikenaka K, Matsuzaki S, Oono K, Katayama T, Tohyama M (2005). Local protein synthesis by BDNF is potentiated in hippocampal neurons exposed to ephrins. Brain Res. Mol. Brain Res..

[22] Paoletti F, Ainger K, Donati I, Scardigli R, Vetere A, Cattaneo A, Campa C (2010). Novel fluorescent cycloheximide derivatives for the imaging of protein synthesis. Biochem. Biophys. Res. Commun..

[23] Pavon-Eternod M, David A, Dittmar K, Berglund P, Pan T, Bennink JR, Yewdell JW (2013). Vaccinia and influenza A viruses select rather than adjust tRNAs to optimize translation. Nucleic Acids Res..

[24] Celma CC, Roy P (2009). A viral nonstructural protein regulates bluetongue virus trafficking and release. J. Virol..

[25] Wirblich C, Bhattacharya B, Roy P (2006). Nonstructural protein 3 of bluetongue virus assists virus release by recruiting ESCRT-I protein Tsg101. J. Virol..

[26] Han Z, Harty RN (2004). The NS3 protein of bluetongue virus exhibits viroporin-like properties. J. Biol. Chem..

[27] Bhattacharya B, Roy P (2013). Cellular phosphoinositides and the maturation of bluetongue virus, a non-enveloped capsid virus. Virol. J..

